# A Benign Rare Lesion of the Breast: Giant Epidermal Inclusion Cyst

**DOI:** 10.7759/cureus.2650

**Published:** 2018-05-18

**Authors:** Ali imran Kucuk, Belma Kocer, Gupse Turan, Yasemin Gündüz, Kayhan özdemir

**Affiliations:** 1 Department of General Surgery, Sakarya University Medical Faculty, Kocaeli, TUR; 2 Department of General Surgery, Sakarya University, Kocaeli, TUR; 3 Department of Pathology, Sakarya University, Kocaeli, TUR; 4 Department of Radiology, Sakarya University, Kocaeli, TUR

**Keywords:** epidermal cyst, breast, surgery

## Abstract

An epidermal inclusion cyst can be seen at any location. Epidermal cysts are commonly found on the scalp, face, trunk, neck, and extremities. They are rarely seen in the breast parenchyma. These benign lesions are important in that they may undergo neoplastic differentiation, although very rarely. Epidermoid cysts usually develop as a result of the implantation of superficial epidermal tissue into the dermis or subcutaneous tissue after trauma or surgical procedures. In this study, a 37-year-old female patient who underwent a histopathological examination that showed a 10-cm epidermal cyst without a history of trauma or a surgical procedure was discussed.

## Introduction

Epidermal cysts are cystic tumors surrounded by keratinized squamous epithelium and filled with keratin remnants [1]. They can be seen in any location. Epidermal cysts are commonly found on the scalp, face, trunk, neck, and extremities. They are rarely seen in the breast parenchyma. These benign lesions are important in that they may undergo neoplastic differentiation, although very rarely. There are also cases in the literature where epidermal inclusion cysts and squamous cell carcinoma are associated [2]. Epidermoid cysts usually develop as a result of the implantation of superficial epidermal tissue into the dermis or subcutaneous tissue after trauma or surgical procedures [3]. This study discusses the case of a 37-year-old with histopathological examination results of epidermal cysts and without any involvement of breast skin or a history of trauma or surgical procedure.

## Case presentation

**Figure 1:**
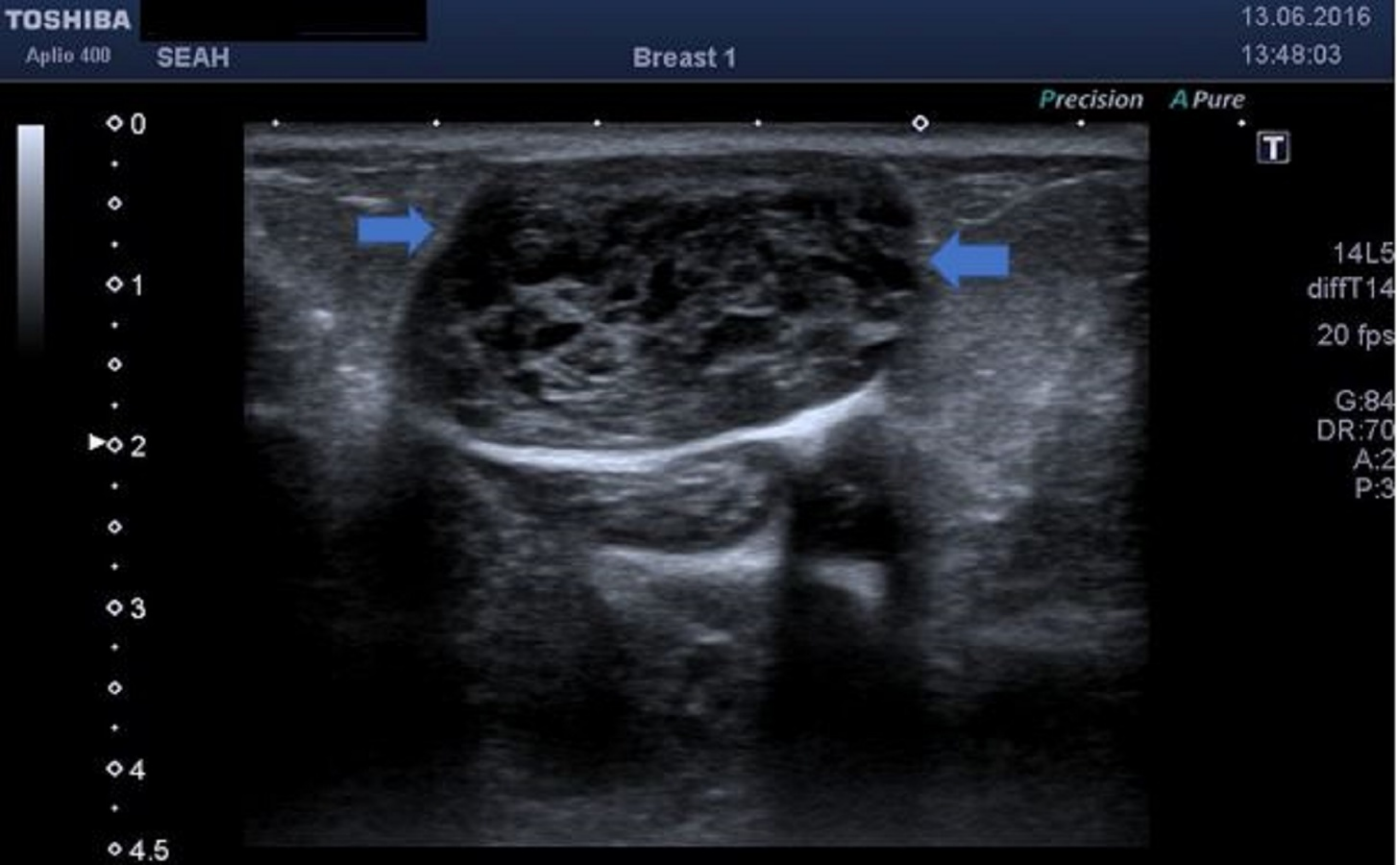
The ultrasonographic view of the cystic area

A 37-year-old multiparous female patient presented to our outpatient clinic with complaints of right breast swelling and stiffness. A physical examination of the patient revealed a well-defined firm mass in the upper outer quadrant of the right breast, approximately 10 x 5 cm in size, extending beneath the areola. An axillary examination of the patient revealed no lymphadenopathy. Breast ultrasonography showed a lobulated, contoured, well-defined, large, hypoechoic multiple solid lesions of heterogeneous pattern in contact with each other in the right breast parenchyma; the largest had a diameter of 7-8 cm and a breast Doppler examination showed no marked vascularization on the mass (Figure 1).

**Figure 2:**
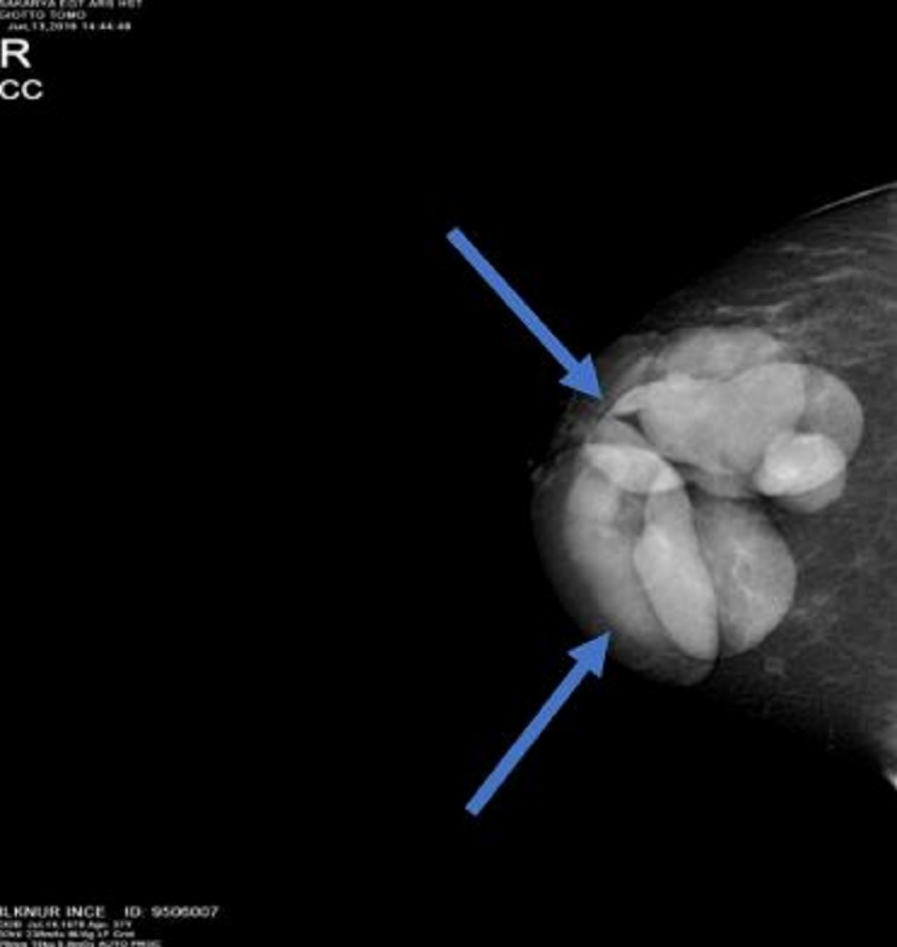
The mammographic image of the cyst; right craniocaudal image

A unilateral mammography was requested. Right breast mammography revealed smooth, contoured, multiple nodular opacities close to the skin without any ductal structure extending to the skin, the largest with a diameter of 6-7 cm (Figure 2) and a bilateral breast magnetic resonance imaging (MRI) was requested.

**Figure 3:**
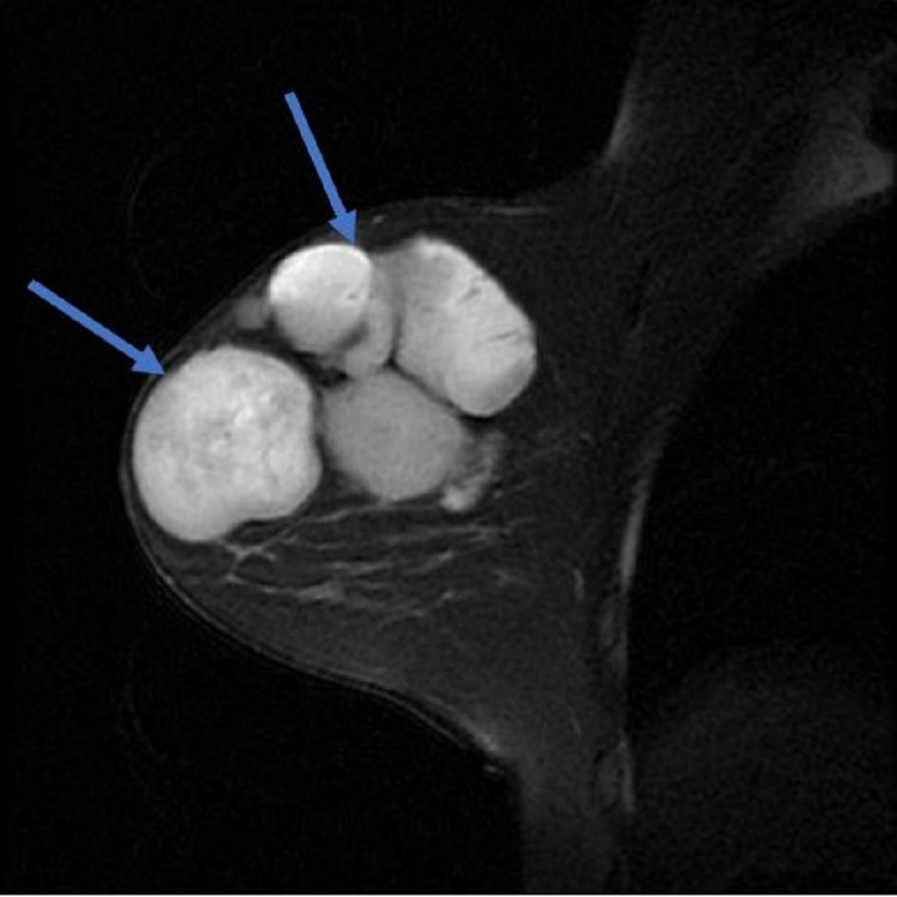
The mammographic image of the cyst; right mediolateral oblique image

**Figure 4:**
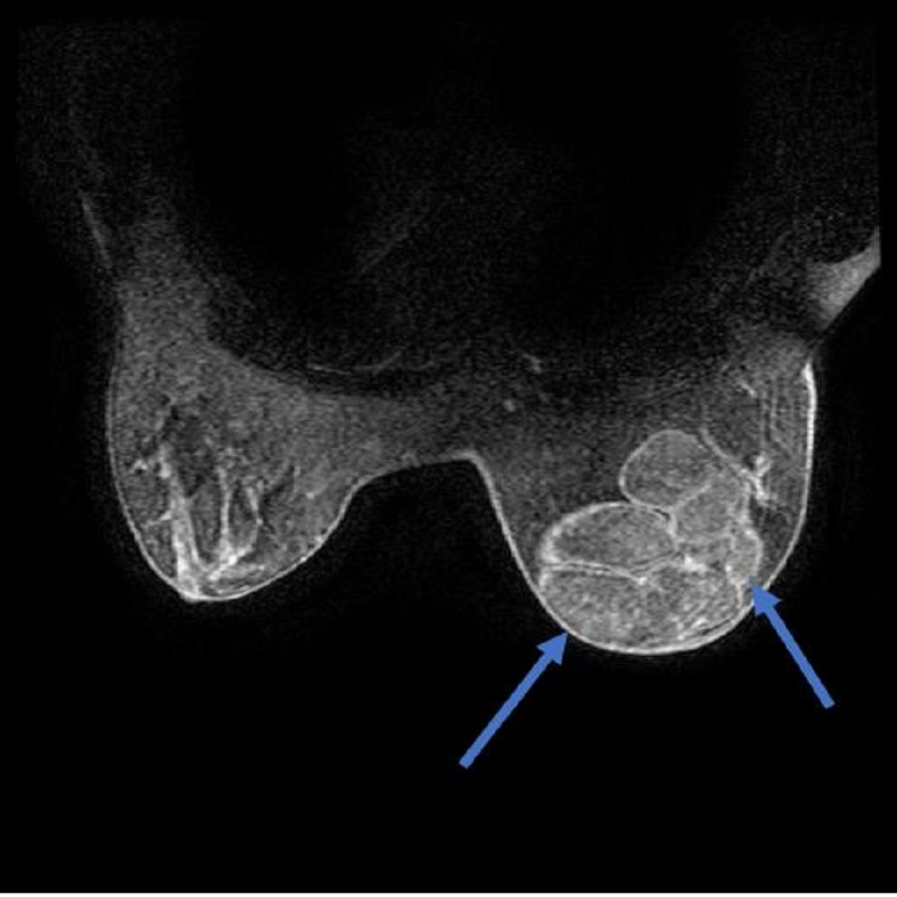
Magnetic resonance imaging of the mass

The breast MRI revealed several, smooth, contoured lesions close to each other, almost completely filling the right breast with equivocal signal feature changes like breast structures, which might partly be consistent with debris and with a complicated appearance; the largest had a size of 7.7 x 4.1 cm. After an intravenous (IV) contrast injection, peripheral contrast enhancement in the cyst wall was observed without marked mural nodular or solid field enhancement at the cyst level. (Figures 3-4).

**Figure 5:**
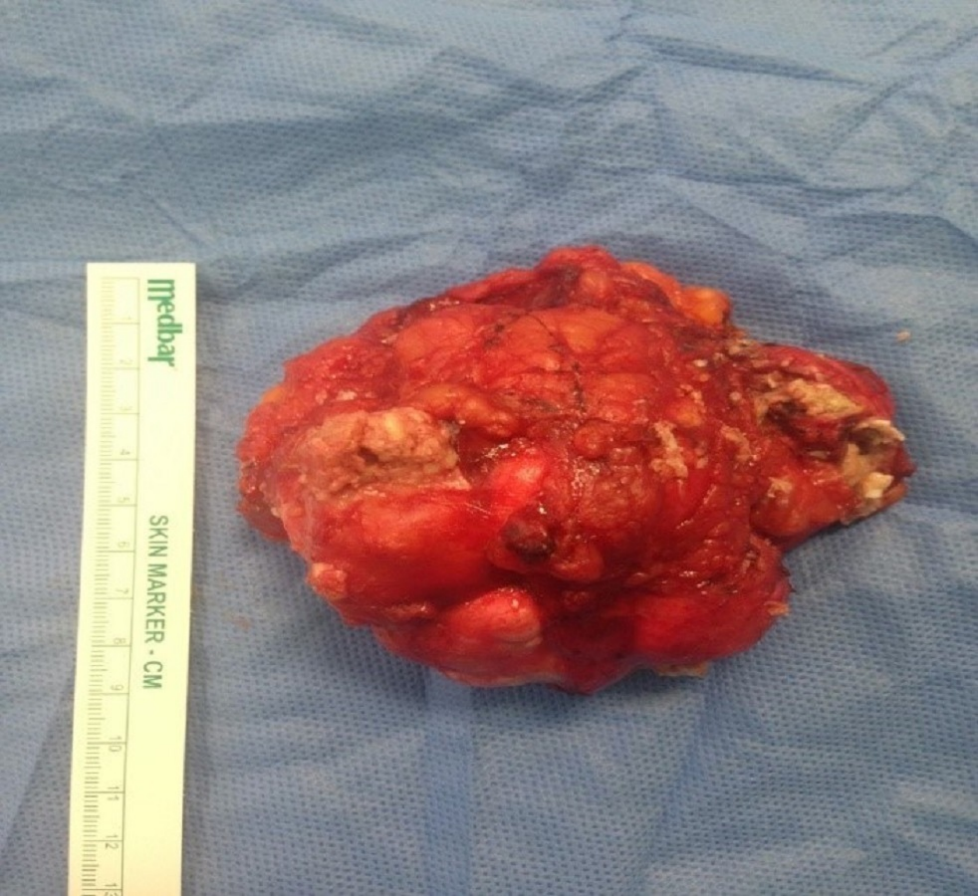
The macroscopic view of the mass

**Figure 6:**
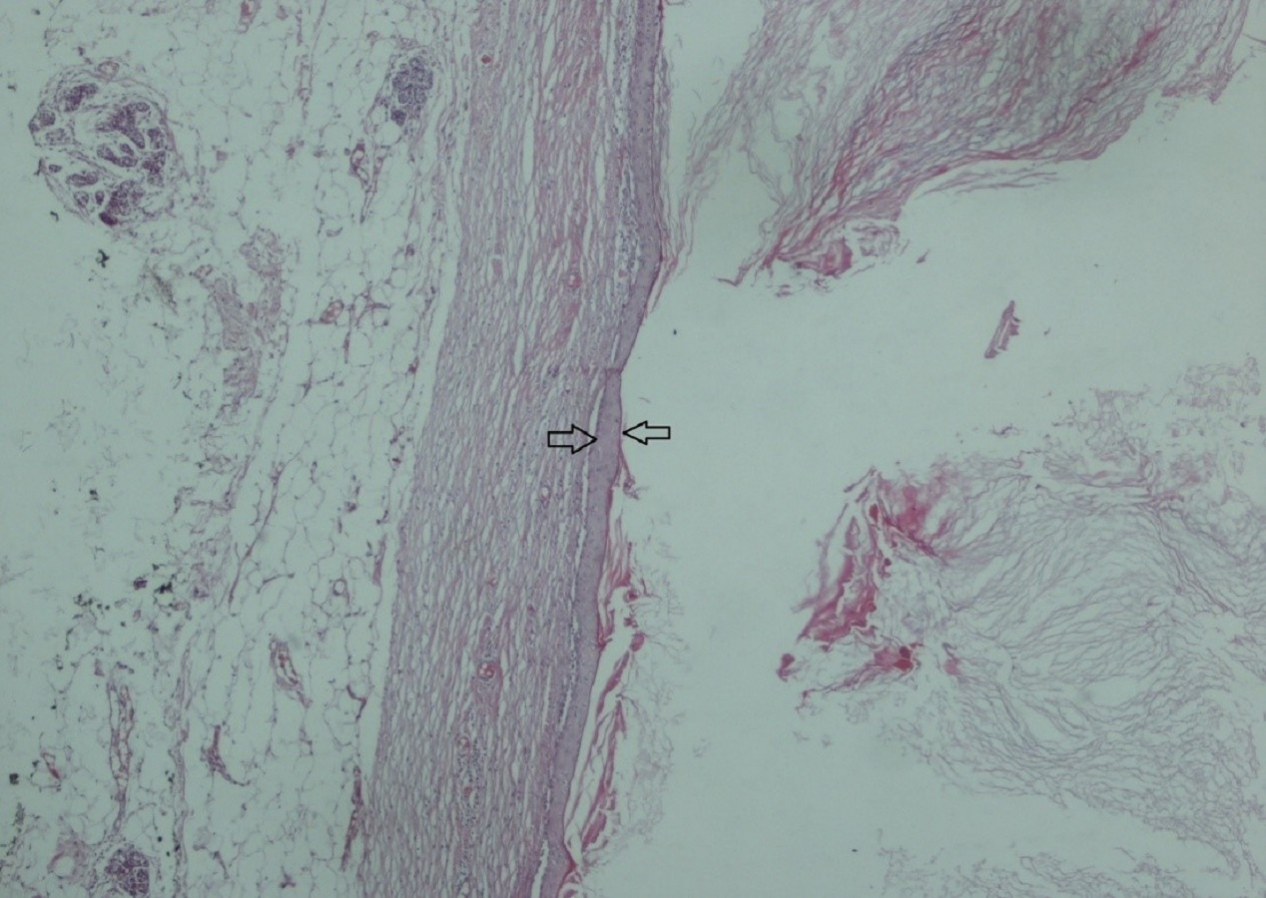
Squamous epithelium fitted (arrow) keratinized material on the lumen facing side and the cystic tissue (H&E X 40) viewed as breast tissue on the other side H&E: hematoxylin & eosin

**Figure 7:**
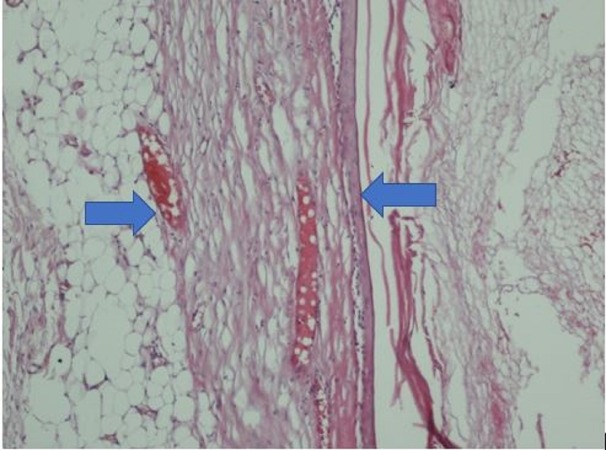
A close image of the lamellar keratin comprising cysts fitted epithelium and cyst content (H&E X 200) H&E: hematoxylin & eosin

A Tru-cut biopsy was performed on the mass. In the pathological examination, benign breast tissue was observed, including tissue fragments lined with keratinized squamous epithelium, keratin materials, and two ductal structures as a separate fragment. Available histomorphological findings were considered consistent with epidermal inclusion cysts. Then, total tumor excision with negative surgical margins and an intraglandular flap reconstruction was performed on May 17, 2017. A post-operative pathological examination yielded epidermal cysts. In the pathological examination, macroscopically, there was a 10-cm-diameter mass with well-defined borders and multiple lobulations (Figure 5); microscopic evaluation reported an epidermal cyst (Figures 6-7).

## Discussion

Epidermal inclusion cysts of the breast parenchyma are rare in the literature [4]. Based on a study by Paliotta et al., a total of 82 cases of epidermal cysts in the breast were reported. In that study, the mean tumor diameter was 3 cm [5]. Our case is one of the rare cases reported in the literature with a diameter of 10 cm.

Several theories were suggested for the development of epidermal inclusion cysts, including congenital occurrence secondary to the obstruction of hair follicles or pores, implantation of epidermal fragments deep into the breast tissue caused by damage to the epidermis after a trauma, and the development of squamous metaplasia in the columnar cells of the dilated ducts in fibrocystic disease, fibroadenomas, and phylloid tumors [4,6]. Our case had no history of any trauma or operation. A mammographic examination of breast epidermal inclusion cysts showed a well-defined appearance with homogeneous densities [4]. The ultrasonography examination showed a solid, well-defined, and complex or heterogeneous appearance. Crystal and Shaco-Levy et al. described the onion ring appearance, a specific ultrasonographic marker of epidermal inclusion cysts of the breast [7]. Ultrasonographically, epidermal inclusion cysts in the breast can often mimic fibroadenomas, phylloid tumors, and mucinous carcinoma, which is a malignant breast lesion with a benign appearance.

Various complications may develop in epidermal inclusion cysts. Epidermal inclusion cysts are benign lesions, but they can rarely transform into squamous cell carcinoma. In a study, the rate of transformation was found to be 19%, but this rate seems too high [8]. A more recent study showed that malignant transformation in the cyst wall epithelium was a very rare condition (0.045%) [2]. There is no definite consensus on the risk of malignant transformation. Other complications are the rupture and risk of infection of the tumor [9].

## Conclusions

In symptomatic patients with large-sized tumors, as in our case, surgical excision of the tumor should be recommended due to the risk of malignant transformation or complication, although the risks are low. However, surgical treatment is not required for cases with asymptomatic, small-sized, stable lesions with a definitive diagnosis of an epidermal inclusion cyst based on imagining techniques and biopsy results.
